# Stem Cells and the Endometrium: From the Discovery of Adult Stem Cells to Pre-Clinical Models

**DOI:** 10.3390/cells10030595

**Published:** 2021-03-08

**Authors:** Lucía de Miguel-Gómez, Sara López-Martínez, Emilio Francés-Herrero, Adolfo Rodríguez-Eguren, Antonio Pellicer, Irene Cervelló

**Affiliations:** 1 IVI Foundation, Health Research Institute La Fe, 46026 Valencia, Spain; lucia.demiguel@ivirma.com (L.d.M.-G.); sara.lopezma@ivirma.com (S.L.-M.); emilio.frances@ivirma.com (E.F.-H.); adolfo.rodriguez@ivirma.com (A.R.-E.); 2 Department of Pediatrics, Obstetrics, and Gynaecology, School of Medicine, University of Valencia, 46010 Valencia, Spain; antonio.pellicer@uv.es; 3 IVIRMA Rome Parioli, 00197 Rome, Italy

**Keywords:** endometrium, niche, stem cells, animal models, regeneration

## Abstract

Adult stem cells (ASCs) were long suspected to exist in the endometrium. Indeed, several types of endometrial ASCs were identified in rodents and humans through diverse isolation and characterization techniques. Putative stromal and epithelial stem cell niches were identified in murine models using label-retention techniques. In humans, functional methods (clonogenicity, long-term culture, and multi-lineage differentiation assays) and stem cell markers (CD146, SUSD2/W5C5, LGR5, NTPDase2, SSEA-1, or N-cadherin) facilitated the identification of three main types of endogenous endometrial ASCs: stromal, epithelial progenitor, and endothelial stem cells. Further, exogenous populations of stem cells derived from bone marrow may act as key effectors of the endometrial ASC niche. These findings are promoting the development of stem cell therapies for endometrial pathologies, with an evolution towards paracrine approaches. At the same time, promising therapeutic alternatives based on bioengineering have been proposed.

## 1. Introduction

Stem cells are undifferentiated cells capable of simultaneously self-renewing and differentiating into multiple tissue-specific cell types under appropriate stimuli [1,2]. Traditionally, stem cells can be classified according to their location and differentiation potency. The most potent stem cells are zygotes, classified as totipotent stem cells, which have the ability to generate a whole embryo and extra-embryonic structures. Then, pluripotent stem cells such as embryonic stem cells give rise to the three primary germ layers, namely endoderm, ectoderm, and mesoderm. Induced pluripotent stem cells, generated by the reprogramming of somatic cells, are included in this group. Unipotent stem cells have the narrowest differentiation capability and divide themselves repeatedly into a single cell type. Finally, multipotent cells give rise to specific lineages [3]. Adult stem cells (ASCs), also referred to as somatic stem cells, are a genre of multipotent stem cells located in specific differentiated organs and can differentiate into a limited type of mature cell to maintain tissue homeostasis [2]. The necessary conditions are provided by the specific anatomical location surrounding the ASCs. This microenvironment, called the stem cell niche, gives rise to autocrine, paracrine, and systemic signals that enable stem cell maintenance and differentiation into specific cell types that participate in tissue repair or regeneration [4,5]. Most of the described ASCs reside in the bone marrow, but they are also detected in several organs and play a crucial role in tissue homeostasis, renewal, and repair [6]. Consequently, this transdifferentiation capacity enables research into therapeutic approaches in tissues such as the blood [7], intestine [8], skin [9], muscle [10], brain [11], and endometrium [12].

The endometrium is the innermost lining of the uterus and its main function is preparing for implantation and attracting the blastocyst towards the uterus. The human endometrium is divided into two different layers with different properties. First, the functionalis, which is the upper layer, is formed by luminal epithelium and subjacent stroma as well as microvasculature. Second, the basalis is constituted by glands and stroma that are preserved throughout the female’s life [13]. During menstruation, the functionalis layer is removed from the body through the menstrual blood, while the basalis remains as an endometrial supply for regeneration of a new functional layer in the next cycle. The human endometrium has some features including menstruation that make it physiologically unique from the murine models. The human menstrual cycle consists of three stages: growth, differentiation, and shedding, which occur around 400 times until menopause [14]. In the mouse, the estrous cycle is divided into four stages (proestrus, estrus, metestrus, and diestrus) occurring every 4 to 5 days, but does not involve menstruation and regeneration of the functionalis layer [15]. Mice undergo up to 80 estrous cycles and can give rise to 8-10 litters during their reproductive life, so active repair and regeneration mechanisms in the endometrial mucosa are critically important [16]. This pronounced remodeling ability and the later proliferative changes seen in adult mammals have driven the hypothesis that there is a niche of ASCs in the endometrium that is activated in every cycle [17,18]. Accordingly, alterations in this endogenous niche could be responsible for endometrial pathology, causing fertility problems [19]. Endometrial pathologies such as Asherman syndrome (AS), caused by the presence of intrauterine adhesions [20], or endometrial atrophy (EA), characterized by an atrophic and usually thin endometrium [21], could originate from insufficient production of endogenous cells and/or a non-functional ASC niche. Other gynecological pathologies such as endometriosis could also be caused by variations in endogenous endometrial ASC activity [19].

**Figure 1:**
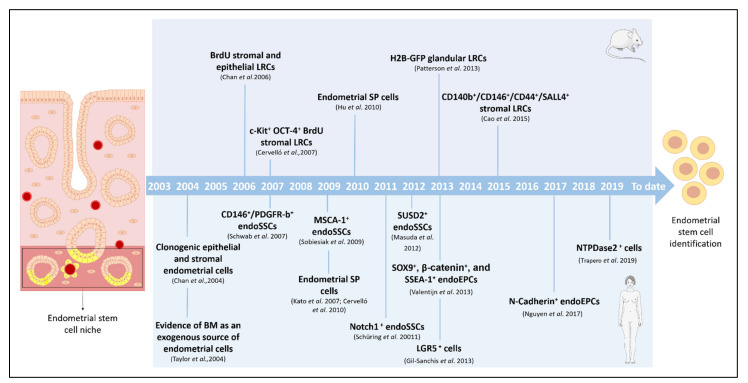
Milestones in the identification, isolation, and characterization of endometrial stem cells. Timeline of the principal findings concerning endometrial stem cells and specific isolation techniques in murine and human models. BM: bone marrow; BrdU: bromodeoxyuridine; CD: cluster of differentiation; endoSSCs: endometrial stromal stem cells; endoEPCs: endometrial epithelial progenitor cells; GFP: green fluorescent protein; H2B: histone 2B; LRCs: label-retaining cells; LGR: leucine-rich repeat containing G-protein-coupled receptor; MenSCs: menstrual blood stem cells; NTPDase2: ectonucleoside triphosphate diphosphohydrolase-2; SSEA-1: stage-specific embryonic antigen-1; SP: side population; SUSD2: sushi domain-containing-2.

Even though endometrial ASCs have been described in different mammals, such as cows [22], pigs [23], sheep [24], horses [25], and non-human primates [26], in this review we focus on the discovery of stem cells in mice and humans, pointing out how these ASCs were identified and describing the different techniques employed (Figure 1). In addition, we highlight the value of pre-clinical models of stem cell therapy to treat AS and EA.

## 2. Endometrial Stem Cells and Specific Niches

In 1978, Schofield proposed the concept of a stem cell niche for the first time, referring to an anatomically defined compartment in which stem cells reside and are able to renew and/or remain undifferentiated [27]. We know now how the niche is specialized and the dynamic microenvironment in which stem cells interact with differentiated cells, secreted factors, and/or components of the extracellular matrix (ECM) [28]. These stimuli determine the behavior (rate and pattern of division) of the stem cells in each specific tissue [29]. Prianishnikov was the first to propose the existence of ASCs in the endometrium [30] as an immature hormone-independent population, residing in the deepest basalis layer with the capability of differentiating towards hormone-responsible endometrial cells. The constant maintenance of this layer along the menstrual (or estrous) cycle makes it a reasonable candidate to be a reservoir of ASCs, even if some stem cells are also found in the functionalis [31]. In 1991, Padykula claimed that the population of stem-like cells migrates and produces different progenitor cells that differentiate specifically into vascular, epithelial, or stromal compartments, thanks to their specific niches [32]. Over the years, these endogenous endometrial ASCs have been proposed not only to be responsible for the cyclic endometrial growth and, consequently, for certain gynecological disorders, but also as useful in therapeutic approaches. According to the origin of the stem cells, endometrial stem cell niches include epithelial, stromal, and endothelial cells, and are likely to contribute to endometrial regeneration [33].

### 2.1. Identifying Endometrial Stem Cells in Murine Models

Our current knowledge about ASCs in the human endometrium would not be possible without reproductive biology studies in animal models, mainly murine ones. Given the absence of specific mouse endometrial stem cell markers, label-retention assays provided critical tools to identify, characterize, and localize these cell populations in vivo (Table 1).

#### 2.1.1. Label-Retention Methods in Murine Models

##### Bromodeoxyuridine Label-Retaining Cells

Label-retention assays are widely used to identify slow-cycling cells in multiple tissues. The concept derives from the quiescent or slow-cycling phenotype shared by most adult or somatic stem cells to preserve their proliferative potential and reduce errors during DNA duplication. The assay consists of the delivery of a pulse of a DNA analog, such as bromodeoxyuridine (BrdU), followed by a chase period in which the analog is absent. This method is not applicable for humans, but is very useful in animal models. When mice are injected with BrdU, all proliferating cells are marked, but only the quiescent ones maintain BrdU during the chase (or a period of time), and are identified as label-retaining cells (LRCs) [76]. This technique identified LRCs in the mouse endometrium [34,35,36,37,38,39,40] to unveil the biology of this tissue and pathologies causing infertility problems.

(1) Stromal BrdU-LRCs

Between 2006 and 2007, two independent studies described the presence of LRCs in the mouse endometrium for the first time [34,35]. After 12 weeks of BrdU injection, Chan et al. identified a small population of stromal LRCs (6%) at the endometrial–myometrial junction, beneath the luminal epithelium, or in a perivascular location near CD31^+^ cells. These LRCs did not express stem cell antigen 1 (SCA-I) or the cluster of differentiation (CD) 45 [77], indicating a non-hematopoietic origin and demonstrating that they were not infiltrating leukocytes. Nevertheless, some LRCs expressed alpha-smooth muscle actin (α-SMA) and estrogen receptor alpha (ER-α) (16%), suggesting they were perivascular cells and responsive to hormonal stimulation.

Moreover, after 8-10 weeks of BrdU injection, Cervelló et al. identified a stromal LRC population expressing the stem cell factor receptor c-Kit and the pluripotent stem cell marker octamer-binding transcription factor 4 (OCT-4), also known as POU5FI. Expression of c-Kit and OCT-4 was restricted to cells located in the lower region of the endometrial stroma, representing 0.32% and 0.19% of the LRCs, respectively [35]. A recent study described stromal LRCs expressing PDGFR-b, CD146, CD44, CD90, and sal-like protein (SALL4) after 6 weeks of BrdU injection. Furthermore, the stromal LRCs persisted during pregnancy and proliferated after delivery, returning to their quiescent status after postpartum repair [36].

(2) Epithelial BrdU-LRCs

In contrast to stromal LRCs, epithelial LRCs have a short persistence in the endometrium of postnatal and prepubertal mice. After 3-4 weeks of BrdU injection, the presence of epithelial LRCs is residual due to greater proliferation of epithelial cells with the initiation of the estrous cycle [34,35]. Chan et al. reported that the epithelial LRCs were mainly located in the luminal epithelium, with cells rarely observed in the glandular epithelium. These cells did not express the leukocyte marker CD45, hematopoietic stem cell antigen SCA-I, or ER-α. This contrasts with the proliferative capacity of epithelial LRCs in response to estrogen, suggesting the existence of indirect stimuli by neighboring ER-α^+^ cells [37].

##### Histone 2B-Green Fluorescent Protein-Label-Retaining Cells

Notably, label-retention assays do not define stemness. Consequently, conclusions can only be drawn about patterns of cell division and tissue regeneration. Therefore, evaluating the functional properties of the identified LRCs is essential.

Recently, the transgenic histone 2B-green fluorescent protein (H2B-GFP) system has allowed us to overcome the limitations of the BrdU label-retention system, since it allows us to isolate viable LRCs. In the H2B-GFP system, cell labeling is done through antibiotic-induced expression of GFP-labeled histones. Two independent studies identified stromal LRCs after using the H2B-GFP system for 3–8 weeks during embryonic development, early postnatal stages, and adulthood [42,43]. Wang et al. reported the presence of epithelial LRCs in the endocervical transition zone and the distal oviduct after 9–13 weeks when the expression of H2B-GFP was induced during embryonic development and until 21 days post-natal [42]. Patterson et al. identified glandular LRCs after 8 months in mice labeled at the peripubertal stage that persisted through various pregnancies. However, this effect was not observed when adult mice were labeled, suggesting that some glandular development is completed the in peripubertal stages [43].

Several studies based on LRC assays showed a higher rate of renewal of the luminal epithelium compared to the glandular (basalis) and stromal fractions of the murine endometrium [38,39,43]. This higher turnover during development facilitates cell labeling and subsequent dilution. These findings are consistent with the idea that the glandular epithelium or a population of stromal stem cells replenishes the luminal epithelium. This added to the identification of stromal LRCs close to the luminal epithelium [34] and led to the proposed existence of a “mesenchymal-to-epithelial transition” phenomenon [39,43]. Likewise, the perivascular distribution of other stromal populations of LRCs [76] is reminiscent of perivascularly located mesenchymal stem cells (MSCs) in humans (see Section 2.2.1), and these LRCs could be involved in regeneration of the vascular stroma [40].

#### 2.1.2. Other Approaches to Identify Murine Endometrial Stem Cells

##### Side Population Cells

ASCs can be identified by differential staining based on the efflux of fluorescent vital dyes, such as Hoechst 33342, thanks to the presence of special ATP-binding cassette (ABC) transporters. Dual-wavelength flow cytometric analysis of these cells reveals a “side population” (SP) [78]. Unlike the findings in humans [44,45,46,47,48,49,50], no SP cells have been identified in the normal cycling endometrium of mice. However, Hu et al. reported the existence of a SP in the postpartum endometrial stroma [51]. This population did not express endothelial, hematopoietic, or mesenchymal stem cell markers, but some cells were positive for SCA-1, c-Kit, and ER-α. Furthermore, based on ER-α expression, these authors suggested the possibility that the SP could differentiate in vitro into tissue-specific endometrial cells under the effect of estrogens.

##### Progenitor Cell Markers in the Mouse Endometrium

Several types of stem cells, such as mesenchymal, hematopoietic, and cancer cells, express the transmembrane protein CD44 [68]. In 2013, Janzen et al. reported that CD44^+^ epithelial cells compose an epithelial progenitor population [52]. In addition to being ER-α and progesterone receptor negative and surviving hormonal deprivation, these cells generate more gland-like structures than do CD44^-^ cells in immunosuppressed mice. In a more recent study, Daene et al. used mice containing a GFP reporter under the control of the telomerase reverse transcriptase promoter (mTert-GFP) to identify potential stem cells in the endometrium [41]. They detected mTert-GFP^+^ populations from epithelial, hematopoietic, and endothelial lineages. Interestingly, mTert-GFP expression did not predict a label-retaining or proliferative phenotype, indicating that these cells are distinct from the previously described slow-cycling cells.

### 2.2. Identifying Endometrial Stem Cells in Humans

In humans, the presence of stem cells in the endometrium was first evidenced in 2004 by two different research groups, both reporting the existence of clonogenic endometrial cells (epithelial and stromal fractions) with high proliferative potential [69] and the specific role of bone marrow-derived stem cells (BMDSCs) in the regeneration of this tissue [70]. As is the case with murine models, due to the lack of well-described specific markers, the first approaches to isolate and identify human endometrial stem cells focused on verification of the universal definition of stemness: self-renewal and differentiation. Differentiation capability is usually demonstrated by multilineage differentiation (usually adipogenic, chondrogenic, and osteogenic lineages) and in vivo tissue reconstruction assays [33]. The gold-standard techniques to demonstrate self-renewal are the demonstration of clonogenicity and long-term culturing capabilities. Other common approaches include searching for cell-surface markers expressed in other known stem cells, such as immunoglobulins or clusters of differentiation. These approaches use cell sorting techniques to select the cell subpopulation of interest. The most common cell sorting techniques are fluorescence- and magnetic-activated cell sorting, referred to as FACS and MACS respectively [71]. The previously described SP method is also commonly used. These techniques (see Table 1) have been implemented in the search for different types of human endometrial stem cells: stromal, epithelial progenitor, and endothelial stem cells. These cells are detected not only in situ, in the endometrium, but also in the menstrual blood. The bone marrow has been revealed as an exogenous source of stem cells contributing to the endogenous endometrial stem cell niche. Thus, when studying this type of stem cell in depth, reference is usually made to two different sources of endometrial stem cells: endogenous and exogenous.

#### 2.2.1. Endogenous Endometrial Stem Cells

In general, three different types of endogenous ASCs (stromal, epithelial progenitor, and endothelial cells) are described in the human endometrium. Any of these endogenous ASC types in the human endometrium could be the cause of endometrial dysfunction.

##### Endometrial Stromal Stem Cells

Like almost all human tissues, the human endometrium contains a small resident population of stromal stem cells (endoSSCs). Typical markers of the mesenchymal phenotype are CD44, CD73, CD90, CD105, and CD106. The absence of other markers such as CD34 or CD45 is also indicative of this type of stem cell [72]. In the endometrium, endoSSCs are located adjacent to the endothelial cells in the microvessels within both the basalis and functionalis layers, suggesting they are also discarded through menstruation [53,54,73]. Several candidates for endoSSCs have been studied in the human endometrium, including the CD146/platelet-derived growth factor receptor beta (PDGFR-b) marker [54], sushi domain-containing-2 (SUSD2) marker [55], SP method [31,44,45], leucine-rich repeat containing G-protein-coupled receptor 5 (LGR5) marker [79], and ectonucleoside triphosphate diphosphohydrolase-2 (NTPDase2) marker [80].

The endoSSCs were first identified as expressing CD146 and PDGFR-b, conventional markers of pericytes [54]. CD146^+^ PDGFR-b^+^ cells express typical markers of MSCs (i.e., CD73, CD90, CD105, and STRO-1) [81] and lack fibroblastic, hematopoietic, and other well-described mesenchymal cell markers, indicating they are perivascular endoSSCs [31,56,82]. To identify a single marker for endoSSCs, human endometrial stromal cell fractions were screened by flow cytometry. SUSD2, also referred to as W5C5, permitted a good isolation of endoSSCs by magnetic bead sorting [53,57]. However, co-expression of SUSD2 with CD146/PDGFR-b^-^ was not consistent [83]. On the other hand, a recent article showed that even CD146^+^ pericytes isolated by FACS present a mesenchymal phenotype and limited potential to regenerate the endometrium [58].

Human endometrial SP cells obtained by the SP technique are hormone-independent with an intermediate telomerase activity and MSC phenotype, allowing for the neoformation of human endometrium in vivo [44,45,48,59]. Recently, our group showed that human endometrial SP cell lines could enhance endometrial reconstitution of SUSD2^+^/ICAM^+^ cells in vivo. We hypothesized that the stromal fraction, which participates in the heterogeneous endometrial SP cell fraction, was acting as the stem cell niche [59].

The universal stem cell marker LGR5 was also detected in the perivascular regions of the functionalis layer, suggesting LGR5^+^ cells are a putative stem cell population [60]. However, the weakness of the in vivo endometrial reconstitution together with CD163 expression and a possible hematopoietic origin suggested that these cells had a more macrophage-like phenotype [79]. Alternatively, NTPDase2 has been detected in perivascular SUSD2^+^ cells but not in the rest of the stromal fraction, and thus has been proposed as a marker for endoSSCs, located in the endometrial basal layer [80].

Other relevant candidates for endoSSC markers are mesenchymal stem cell antigen-1 (MSCA-1), also known as tissue non-specific alkaline phosphatase or TNAP [61], and Notch1 [62]. However, their utility is limited as they are not suitable cell markers for endoSSC isolation [84].

Finally, precursors of human decidual stromal cells from decidua-endometrial biopsies, obtained from first-trimester pregnancies, exhibit features compatible with perivascular endoSSCs (also expressing CD146 and PDGFR-b) in addition to the ability to decidualize in vitro, suggesting they are decidual MSCs involved in the mechanisms of maternal–fetal immune tolerance [74].

##### Endometrial Epithelial Progenitor Cells

Endometrial epithelial progenitor cells (endoEPCs) are a subpopulation of cells located at the base of the glands in the basalis layer [63,64,83]. Although some studies have proposed that endoEPCs could derive from endometrial stromal cells [39,85], epithelial stem cells have been identified in the human endometrium. In addition, endoEPCs have been obtained from epithelial SP fractions, presenting the epithelial cell marker CD9 and producing gland-like structures in vivo [44,45].

Stage-specific embryonic antigen-1 (SSEA-1) can also identify the human glandular epithelial cells of the basalis, distinguishing them from those in the functionalis. SSEA-1^+^ endoEPCs are quiescent, with long telomeres, and can produce endometrial gland-like spheroids in three-dimensional (3D) in vitro culture [63]. The atrophic menopausal endometrium is also SSEA-1^+^ and regenerates itself to produce a functional layer under estrogen supply, suggesting that endoEPCs last after menopause [55,63,64]. The genes SOX9 and beta-catenin [63] have also been studied as putative cell markers of endoEPCs together with SSEA-1, although they usually require co-detection with other specific markers, and additional studies are needed.

Recently, N-cadherin, a protein previously related to the stem cell niche in other organs [86,87,88,89], has been found in quiescent cells from the deep basalis [63,64]. N-cadherin^+^ epithelial cells are more clonogenic than are N-cadherin^-^/cytokeratin^+^ epithelium and can be differentiated to gland-like organoids. However, N-cadherin did not immunolocalize with SSEA-1 [64]. Nguyen et al. suggested a differentiation pyramid hypothesis initialized by the higher abundance of immature N-cadherin^+^/SSEA-1^−^ endoEPCs at the base of the glands, adjacent to the myometrium. As these primitive N-cadherin^+^/SSEA-1^−^ cells move through the basalis, they gradually lose N-cadherin expression and differentiate into transit N-cadherin^-^/SSEA-1^+^ cells, which completely differentiate when they arrive at the functionalis [64]. However, more functional studies are required to clarify this hypothesis.

Finally, the presence of NTPDase2 in surrounding basal glands, but not in functional glands, could be linked to the putative action of NTPDase2 in the preservation of endoEPCs [80].

##### Endothelial Stem Cells

Endothelial stem cells have also been postulated to be part of the endometrial stem cell niche. These cells are CD31^+^/CD34^+^ (classical endothelial markers) and have been detected among the SP endometrial cells (isolated using the ATP-binding cassette subfamily G member 2 or ABCG2 marker) located not only in the basalis but also in the vascular endothelium [47]. This heterogeneous endometrial subpopulation of SP cells also has a higher expression of endothelial markers than do non-SP cells [48].

#### 2.2.2. Exogenous Endometrial Stem Cells

The bone marrow, which contains a heterologous cell population including progenitors, hematopoietic, and non-hematopoietic stem cells [90], is proposed as an external source of ASCs, contributing to the endometrial stem cell niche.

##### Contribution of Bone Marrow to the Endometrial Stem Cell Niche

BMDSCs are MSCs that serve as an exogenous source of stem cells. BMDSCs are found in several tissues and have the capability to repopulate non-hematopoietic tissues. Some authors postulate that these BMDSCs could shift towards the endometrial stem cell niche and differentiate into tissue-specific stem cells [70]. In both human and mouse models, BMDSCs are able to differentiate into non-hematopoietic endometrial cells such as epithelial, stromal, and endothelial cells [65,66,70]. Cervelló et al. confirmed BMDSCs migrate towards the endometrium and contribute to the cell composition of the stroma and the epithelial compartment in patients receiving bone marrow transplants, although bone marrow is proposed to be a limited exogenous source rather than a cyclic source of BMDSCs [50]. The contribution of this exogenous source of stem cells to the endothelial cells after angiogenic pro-regenerative events was also reported [91].

Additionally, there is no agreement on established markers and their characterization due to the complexity of the endometrium and the high likelihood of coexistence of several niches in other tissues [92]. In general, MSCs express varied cell surface markers such as CD29, CD44, CD73, CD90, and/or CD105 and do not express CD14, CD34, CD45, and/or HLA-DR, nor is the expression of these epitopes clearly observed during transdifferentiation processes in other tissues [93]. Clonogenic endometrial cells from humans show similar properties in vitro to those of mesenchymal stem cells [44], thus making it questionable whether these identifying properties are valid or not [83].

Consequently, the contribution of this source of stem cells in the endometrium is still under debate: It is unsettled whether they have a role in the remodeling process or if they just show a uterine phenotype after migrating towards the endometrium [83]. Nevertheless, the exogenous source offers great potential for treatment of infertility caused by endometrial alterations.

### 2.3. Role of Stem Cells in Endometrial Pathologies

Stem cells can participate in the development of infertility-causing diseases directly related to the uterus and/or endometrium, such as endometrial hyperplasia, endometrial carcinoma, endometriosis, adenomyosis, and leiomyomas.

Endometrial hyperplasia occurs when the endometrial lining is too thick, and it is a risk factor for endometrial carcinoma, characterized by endometrial epithelial neoplasia [75]. In this type of carcinoma, endogenous ASCs are thought to transform into cancer stem cells (CSCs) through a series of genetic alterations and/or epigenetic mutations, which could be responsible for tumor formation [75]. Additionally, a SP subpopulation was isolated from endometrial carcinoma samples [94], while CD44, CD55, and CD133 were used to isolate these CSCs [95].

The role of stem cells has also been suggested in the pathogenesis, though still unknown, of endometriosis [96], implying the presence or development of endometrial tissue in ectopic locations, and adenomyosis [97], characterized by the presence, focalized or diffused, of ectopic endometrial tissue in the myometrium. In endometriosis, a multifactorial disease, three main hypotheses concerning ASCs have been postulated [31]. The first, and most accepted one, is Sampson’s theory of retrograde menstruation. This hypothesis implies that putative menstrual blood stem cells arriving at ectopic locations are the origin of the endometriotic lesions [96]. The second one is that endometriosis in prepubertal girls is explained by endometrial ASCs from neonatal uterine bleeding caused by maternal hormone withdrawal right after delivery [98]. The third and last theory is that the relationship between endometrial stem cells and progesterone resistance of the endometrial stromal cells impairs their ability to decidualize [99]. Whatever the mechanism by which the endometrial stem cells arrive at and generate endometriotic lesions, these stem cells can express different stem cell markers such as SEEA-1 and N-cadherin [31].

Migration of menstrual blood stem cells from retrograde menstruation into the peritoneal cavity could also generate focal adenomyosis [97]. Further, endometrial ASCs in the basalis layer of patients with adenomyosis present more pseudopods and thus may more easily migrate towards the endometrium, generating adenomyotic lesions [100].

## 3. Stem Cell Therapy and the Endometrium: The Importance of Basic Research and Pre-Clinical Models

The knowledge acquired during the search for an endogenous endometrial stem cell population has contributed to stem cell therapy, widely used in other medical fields, for endometrial pathologies. Stem cell therapy is based on the capacity of the stem cells to arrive at the damaged site, self-renew, and differentiate into target tissue cells to empower the repopulation and regeneration of, in this case, the endometrium. However, with the growing implementation of stem cell therapies and low engraftment of the cells observed in some studies [101,102], other mechanisms of action have been proposed.

### 3.1. Paracrine Action of Stem Cells: Main Findings in the Endometrium

Currently, the paracrine hypothesis is probably the most documented and accepted one. Following this premise, stem cells secrete different biomolecules such as growth, angiogenic, or immunosuppressive factors, as well as chemokines and exosomes, which contribute to regeneration of the injured tissue [103]. In line with these secreted immunosuppressive factors, the stem cells are also thought to have an immunomodulatory potential to manage the inflammation status of the injured site and prepare the tissue for the incoming regenerative events [104]. Different studies in animal models corroborate this paracrine hypothesis by elucidating low stem cell engraftment when applied to a damaged endometrium [105]. In addition, this therapy has similar effectiveness whether it is administered through the tail vein or directly into the uterine horns [105,106]. These findings support the hypothesis that the regenerative potential of stem cells might not be due to proliferation of the administered stem cells themselves but to the paracrine factors they secrete or stimulate in the receptor organism. Interestingly, injected stem cells engraft into the spiral arterioles of the endometrium where the stem cell niche is thought to be located [107]. This finding reinforces the existence of this stem cell niche and the paracrine effect that the endogenous cells exert to attract the therapy (stem cells in this case) to directly act over the niche and promote tissue regeneration.

The immunomodulatory action of these stem cells in the target tissue was also elucidated in the endometrial milieu. Downregulation of some genes such as chemokine *CXCL8* is hypothesized to reduce the inflammation status and induce the expression of other factors such as *SERPINE1* or proto-oncogene *c-JUN*, preparing the tissue for the incoming regeneration processes [107].

### 3.2. Stem Cell Therapy for Treating Endometrial Pathologies

To treat AS and EA, different sources of stem cells have been used. The most explored sources for endometrial regeneration are the umbilical cord [108,109], amniotic membrane [110,111], bone marrow and adipose tissue [112], in addition to menstrual blood [113], or even autologous endometrial biopsies [114,115]. These last four types of stem cells usually have an autologous origin. Thus, complications such as graft-versus-host disease, associated with allogenic stem cell transplant, do not apply; however, they can involve other disadvantages such as an insufficient number of cells [116]. This could explain why most published works in the field, most of them in the form of pilot studies or case reports, directly try the therapy in humans [117]. However, using pre-clinical models, usually rodents (mice and rats), mimicking either the adhesions and fibrotic tissue characteristic of AS patients or the thin and/or atrophic endometrium of patients with EA, is important before translating medical approaches to women [118], which is always the final goal. The importance of these animal models resides in their capacity to predict the outcome of future clinical trials.

#### Pre-Clinical Models of Endometrial Injury

Bone marrow is an excellent source for obtaining stem cells to treat endometrial pathologies. The contribution of these stem cells to the endometrial stem cell niche has probably encouraged their use. As mentioned before, the bone marrow contains a heterologous cell population, [90], thus, it is important to distinguish which subpopulation of stem cells is administered into murine models of AS or injured endometrium. Some studies use the non-hematopoietic or stromal BMDSCs that are positive for CD29, CD44, CD73, and/or CD90, but negative for CD45 or CD34 [106,119,120,121]. Other research groups inject hematopoietic BMDSCs cells using different markers such as CD133 antigen [105]. Furthermore, other studies report the use of the whole bone marrow stem cell fractions [122,123].

Studies using other stem cell sources [108,109,110,111,112,113,114,115] to treat animal models of endometrial injury (umbilical cord, adipose tissue, amniotic membrane, menstrual blood, and endometrial biopsies) mainly explored the mesenchymal stem cell type using markers such as CD29, CD44, CD73, CD90, and CD105.

## 4. Future Perspectives and Next Steps

There is still a lack of knowledge in the basic biology of the endometrial stem cell niche and more efforts should be directed to find specific stem cell markers. Understanding the human endometrial stem cell niche is important not only to moving forward in the gynecological field, which is the main objective, but also to potentially progressing other medical areas. The potential of endometrial stem cells, either obtained from the menstrual blood or the endometrial tissue, has been described in other medical fields. Indeed, studies propose the use of endometrial stem cells for treating Parkinson’s disease in primates [124] and a mouse model of encephalomyelitis [125].

However, currently, the trend is in refining stem cell therapy to treat endometrial pathologies in combination with other approaches to enhance its pro-regenerative action. In some studies, chemokines or growth factors are applied in combination with MSCs [67,123]. Other works make use of a very promising biological product in the regenerative medicine field, platelet-rich plasma [126]. Bioengineering approaches such as hydrogels [121] or collagen scaffolds [127] obtained from the ECM have also been explored. The ECM contains a variety of growth factors and other molecules involved in regeneration processes [128]. Finally, in line with the paracrine action hypothesis, derivatives from the stem cells themselves, such as the stem cell secretome [129] or exosomes [130], alone or in combination with other approaches, have also been revealed as promising for restoring the endometrial tissue in murine models of endometrial injury. In parallel, there are also research projects focused on optimizing the isolation of stem cells derived from menstrual blood [131,132], which has emerged as a promising source of stem cells.

## 5. Conclusions

Stem cells are key to the normal functionality of the endometrium. Therefore, complete understanding of their activity is essential in management of endometrial pathologies, not only as of the cause of these disorders, but also as the solution (either as the therapy or the target of other approaches).

As reported here, the presence of endogenous stem cells in the endometrium has been widely described and accepted. However, there is still a lack of standardization in detection and isolation methods for this specific population of ASCs. Future studies will likely find specific stem cell markers or validate those that have already been proposed.

Stem cell identification methods commonly used in animal models, such as the BrdU-based assay, have the major disadvantage of not allowing functional assays due to the required fixation procedure. These assays also differ from the methods used historically in the pursuit of endometrial stem cells in humans. Thus, further studies focused on developing methods for identification of endometrial stem cells in animal models would help to equate and compare findings with the human models and consequently to facilitate clinical translation of incoming therapies. These therapies for endometrial management have evolved along the years from classic stem cell therapy to emerging approaches based on the paracrine activity of the stem cells and bioengineering.

## Figures and Tables

**Table 1:** Endometrial stem cell identification and isolation techniques in human and murine models. All procedures reviewed in the text are summarized and classified.

Identification/Isolation Method		Main Characteristic	Application in Murine Endometrial ASCs	Application in Human Endometrial ASCs	References
Label-retention methods	BrdU	DNA analog Pulse-chase assays	YES	NO	[34,35,36,37,38,39,40,41]
	H2B-GFP	Transgenic system Allows detection of viable cells	YES	NO	[42,43]
Side population		ABC-transporter-positive cell identification	YES	YES	[44,45,46,47,48,49,50,51]
Stem cell marker identification	Flow cytometry	Allows identification and isolation; Compatible with multiple-marker profiles	YES	YES	[41,44,45,47,48,49,51,52,53,54,55,56,57,58,59,60,61,62,63,64,65,66,67]
	MACS	Magnetic labeling-based cell isolation method; Preferable for single marker procedures	NO	YES	[49,53,55,57,61,63,64]
Clonogenicity assays		Evaluates the ability of a single cell to produce a colony	YES	YES	[41,44,45,47,48,49,51,52,53,56,57,61,62,64,68,69,70,71,72,73,74,75]
Long-term culture		Stem cells exhibit long-term proliferative potential	NO	YES	[45,53,62,75]
Multi-lineage differentiation		MSCs can differentiate into adipocytes, osteoblasts, myocytes, and chondrocytes in vivo and in vitro	YES	YES	[44,45,46,52,53,54,55,56,57,58,62,65,67,74]

ASCs: adult stem cells; BrdU: bromodeoxyuridine label-retaining cells; H2B-GFP: histone 2B-green fluorescent protein; MACS: magnetic-activated cell sorting; MSCs: mesenchymal stem cells.

## Data Availability

Not applicable.
